# Temple-Baraitser Syndrome and Zimmermann-Laband Syndrome: one clinical entity?

**DOI:** 10.1186/s12881-016-0304-4

**Published:** 2016-06-10

**Authors:** André Mégarbané, Rashid Al-Ali, Nancy Choucair, Monko Lek, Ena Wang, Moncef Ladjimi, Catherine M. Rose, Remy Hobeika, Yvette Macary, Ramzi Temanni, Puthen V. Jithesh, Aouatef Chouchane, Konduru S Sastry, Remy Thomas, Sara Tomei, Wei Liu, Francesco M. Marincola, Daniel MacArthur, Lotfi Chouchane

**Affiliations:** Institut Jérôme Lejeune, Paris, France; Bioinformatics Division, Sidra Medical & Research Center, Doha, Qatar; Medical and Population Genetics, Broad Institute of Harvard Medical School, Boston, USA; Genomics Core Laboratory, Translational Medicine Division, Sidra Medical & Research Center, Doha, Qatar; Laboratory of Protein Chemistry, Weill Cornell Medicine-Qatar, Doha, Qatar; POSSUMweb, Victorian Clinical Genetics Service and Murdoch Childrens Research Institute, The Royal Children’s Hospital, Parkville, VIC Australia; Dermatology Research Group, Translational Medicine Division, Sidra Medical & Research Center, Doha, Qatar; Laboratory of Genetic Medicine and Immunology, Weill Cornell Medicine-Qatar, Education City, Qatar Foundation, Doha, Qatar; Research Office, Sidra Medical & Research Center, Doha, Qatar

**Keywords:** Temple-Baraitser syndrome, Whole genome sequencing, *KCNH1*, Zimmermann-Laband syndrome

## Abstract

**Background:**

*KCNH1* encodes a voltage-gated potassium channel that is predominantly expressed in the central nervous system. Mutations in this gene were recently found to be responsible for Temple-Baraitser Syndrome (TMBTS) and Zimmermann-Laband syndrome (ZLS).

**Methods:**

Here, we report a new case of TMBTS diagnosed in a Lebanese child. Whole genome sequencing was carried out on DNA samples of the proband and his parents to identify mutations associated with this disease. Sanger sequencing was performed to confirm the presence of detected variants.

**Results:**

Whole genome sequencing revealed three missense mutations in TMBTS patient: c.1042G > A in *KCNH1*, c.2131 T > C in *STK36*, and c.726C > A in *ZNF517*. According to all predictors, mutation in *KCNH1* is damaging *de novo* mutation that results in substitution of Glycine by Arginine, i.e., p.(Gly348Arg). This mutation was already reported in a patient with ZLS that could affect the connecting loop between helices S4-S5 of *KCNH1* with a gain of function effect.

**Conclusions:**

Our findings demonstrate that *KCNH1* mutations cause TMBTS and expand the mutational spectrum of *KCNH1* in TMBTS*.* In addition, all cases of TMBTS were reviewed and compared to ZLS. We suggest that the two syndromes are a continuum and that the variability in the phenotypes is the result of the involvement of genetic modifiers.

## Background

Temple-Baraitser syndrome (TMBTS; MIM: 611816) and Zimmerman-Laband syndrome (ZLS; MIM: 135500) are rare developmental disorders with hypoplasia/aplasia of nails. These syndromes are considered to be distinct entities, with TMBTS defined as a disorder characterized by severe intellectual disability (ID), epilepsy, hypoplasia/aplasia of the nails of the thumb and great toe, a pseudo-myopathic appearance, and marked hypotonia in infancy [1–6], and ZLS charatacterized by ID, gingival fibromatosis, associated with absence or dysplasia of all nails, hypoplasia of the distal phalanges, scoliosis, hepato-splenomegaly, coarse face, and hirsutism [7].

*KCNH1* encodes a voltage-gated potassium channel that is predominantly expressed in the central nervous system, and mutations in this gene have been linked to both syndromes [6, 7].

Here, we report on a Lebanese male patient with TMBTS having a mutation in *KCNH1* that has previously been reported in a patient with ZLS. In addition, we have reviewed all published cases of TMBTS and highlight common features, as well as critical differences, between these two syndromes, and raise the issue of whether their classification into two entities is appropriate.

## Methods

### Clinical report

The male proband is the third child of healthy unrelated Lebanese parents. He was born at 36 weeks of gestation, after a complicated pregnancy characterized by the therapeutic administration, to the mother, of drugs against early contractions at 32 weeks of gestation. At birth, his weight was 2700 g (60^th^ percentile), his length 48 cm (75^th^ percentile) and his head circumference (OFC) 33 cm (60^th^ percentile). Family history was unremarkable. Marked hypotonia, constipation, and aplasia of thumb and great toe nails were noted in the first two to three days of life.

**Fig. 1:**
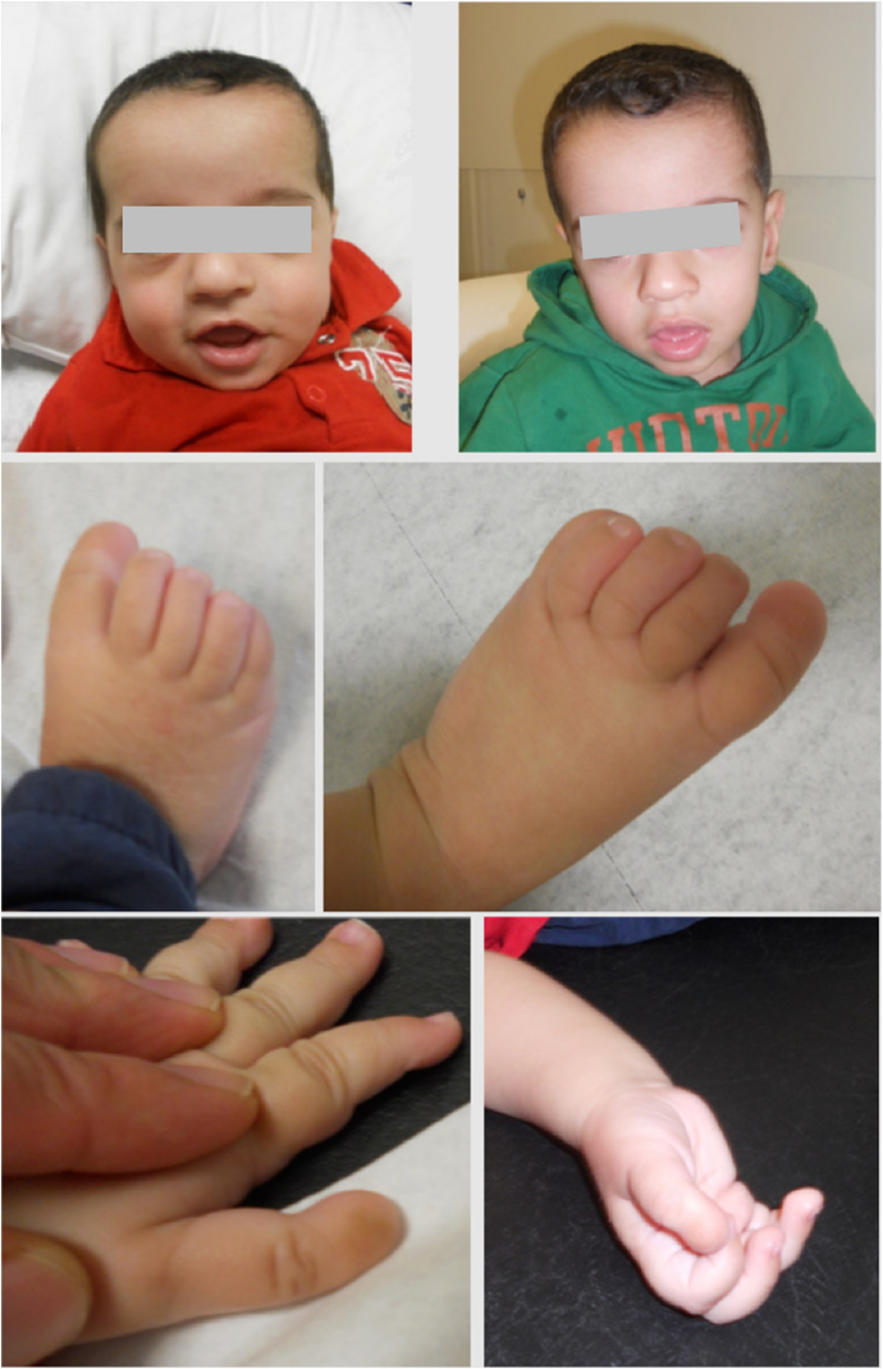
Photographs of the patient at the age of 9 and 15 months. Note the frontal bossing, mild hypertelorism, broad and depressed nasal bridge, broad mouth with downturned corners, full cheeks and the myopathic face. Both thumbs are held in an adducted posture are terminally broad with aplasia of the nails bilaterally. Big toes are also broad, long, with aplasia of nails

The propositus was referred for genetic examination at the age of 9 months. His weight was 9750 g (60^th^ percentile), length 71.5 cm (75^th^ percentile), OFC 42.7 cm (10^th^ percentile). He had a flat occiput, a frontal bossing, large ears, mild hypertelorism, epicanthal folds, a broad and depressed nasal bridge, a short columella, long philtrum, a broad mouth with downturned corners, a high arched palate, 2 upper and 2 lower incisors of normal shape, and full cheeks (Fig. 1). Widely spaced nipples and left chest depression were also noted. Both thumbs were held in an adducted posture and were terminally broad with aplasia of the nails bilaterally. Big toes were also broad, long, and with aplasia of nails. No hirsutism, no hypoplasia of the distal phalanges, no hypermobility, no camptodactyly, nor palmar creases were noted.

**Fig. 2:**
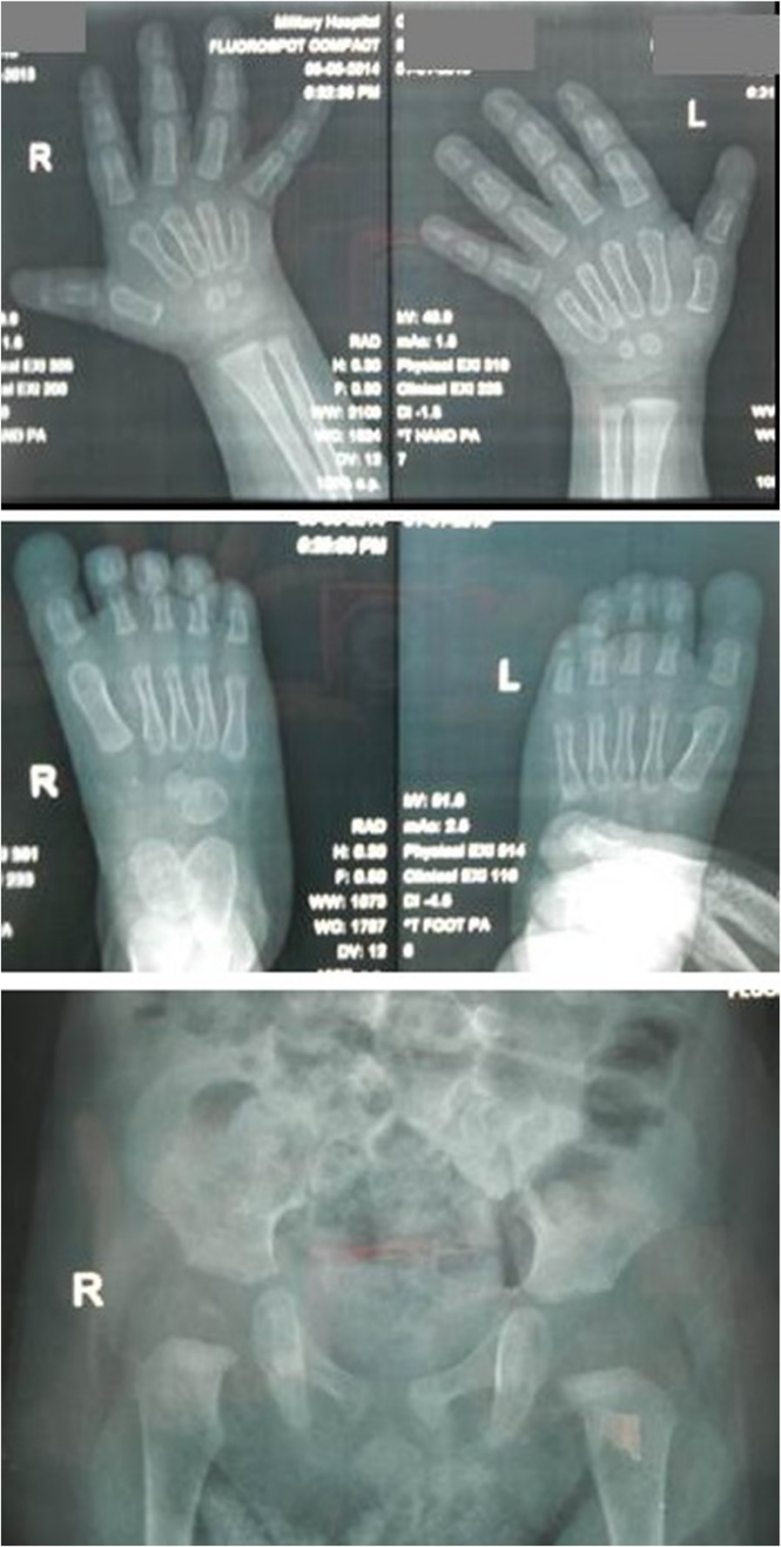
X-ray films of the patient. Note the absent distal phalanges of the thumbs and great toes, and the very small femoral epiphyses at the age of 15 months

At 15 months old, his weight was 11 kg (75^th^ percentile), length 79 cm (50^th^ percentile), and OFC 45.7 cm (10^th^ percentile). Delays in developmental milestones were striking, as he could not stand up alone or walk with help, and could not follow or respond to simple commands. He had a myopathic face with poor visual contact, a wide open mouth and mild gingival enlargement (Fig. 1). Skeletal survey revealed nearly absent distal phalanges of the thumbs and great toes, very small femoral and humeral epiphyses, and an osteosclerosis of the anterior arc of the right 10^th^ rib (Fig. 2).

Magnetic resonance imaging, abdominal and heart ultrasound, brain stem auditory evoked responses, and EEG were normal. Complete blood count, hemoglobin electrophoresis, serum electrolytes, blood glucose levels, urinalysis, thyroid, liver and renal function tests were all unremarkable. Array CGH analysis and Chromosomal Microarray Analysis did not reveal any abnormalities (data not shown).

### DNA extraction and Whole Genome Sequencing (WGS)

Whole genome sequencing was carried-out on the patient and his parents using the HiSeq 2500 sequencer (Illumina, San Diego, CA, USA). Libraries were generated from 1 μg of genomic DNA [8] using the Illumina TruSeq DNA PCR-Free Sample Preparation Kit. Genomic DNA was sheared using the Covaris system (Woburn, MA, USA). Isolated DNA fragment ends were blunted, A-tailed and ligated with sequencing adaptors with index sequences. Excess adapters and enzymes were removed using AMPure beads (Beckman Coulter Genomics, Danvers, MA, USA). Indexed libraries were size selected to 350 bp range using bead-based capture and the concentration of amplifiable fragment was determined by qPCR relative to sequencing libraries with known concentration. Normalized libraries were clustered on a c-BOT machine and 125 bp paired-end sequencing was performed on the HiSeq2500 system.

### WGS data analyses

Raw data was mapped to the human genome reference build 19 (http://www.broadinstitute.org/ftp/pub/seq/references/Homo_sapiens_assembly19.fasta) using BWA aligner [9] version 0.7.7-r441 and variant call was performed using GATK [10] version 3.3.2. The rare variant analysis was performed using the xbrowse tool (https://xbrowse.broadinstitute.org/). For the parents and the child, a ‘De novo Dominant’ inheritance model was selected, with severity of the variant effect set to ‘moderate to high impact’ (Nonsense, essential splice sites, missense frameshift and in frame), call quality as high (genotype quality > 20 and allele balance ratio > 25 %) and allele frequency < 1 % in 1000 genomes and The Exome Aggregation Consortium (ExAC) v0.3 datasets. Functional consequences of amino acid substitutions have been predicted using various tools [11–14].

### Sanger sequencing

Genomic sequences of *KCNH1, STK36,* and *ZNF517* were obtained from UCSC Genome Browser (December 2013). A flanking region around each sequence variant site was amplified by PCR with the following primer pairs: forward primer (5′-TCAACGCTTTTGAGAACGTG-3′) and reverse primer (5′-TGTCTTGGTGTCCTCGTCAA-3′) for *KCNH1* (NM_002238); forward primer (5′-CATCCCTCATCTCTGGCCTG-3′) and reverse primer (5′-ACTTTTACCTTGCCCTGAATCA-3′) for *STK36* (NM_001243313); and forward primer (5′-TTCAAGCAAAGCTCCATCCT-3′) and reverse primer (5′-GGTGTGGAACTTCTGGTGCT-3′) for *ZNF517* (NM_213605). Primers for the PCR amplifications were designed using Primer3 Software. PCR reactions were performed using Taq DNA polymerase (Invitrogen Life Technologies, Carlsbad, CA, USA). PCR fragments were run on 1 % agarose gel. The fragments were purified using the Illustra_ GFX_ PCR DNA and Gel Band Purification Kit (GE Healthcare) and then sequenced using the Big Dye_ Terminator v 1.1 Cycle Sequencing Kit (Applied Biosystems, Foster City, CA, USA). Sequence reaction was purified on Sephadex G50 (Amersham Pharmacia Biotech, Foster City, CA), and then loaded into an ABI 3100 system after the addition of Hidi formamide. Electropherograms were analyzed using Sequence Analysis Software version 5.2 (Applied Biosystems) and then aligned with the reference sequences using ChromasPro version 1.22 (Technelysium, Queensland, Australia).

## Results

Whole Genome Sequencing identified 3 missense mutations in TMBTS patient (Table 1). We validated and confirmed the *de novo* origin of these variants by Sanger sequencing.

**Table 1:** Variants identified with the WGS analysis while running a *de novo* dominant model using xbrowse

Gene	Position	Function	Software prediction
KCNH1	chr1:211093321	Missense	Polyphen: probably damaging
	C > T	c.1042G > A	Sift: damaging
		p.(Gly348Arg)	Mutation taster: disease causing
			Fathmm: damaging
STK36	chr2:219558050	Missense	Polyphen: possibly damaging
	T > C	c.2131 T > C	Sift: damaging
		p.(Cys711Arg)	Mutation taster: disease causing
			Fathmm: tolerated
ZNF517	chr8:146033027	Missense	Mutation taster:disease causing
	C > A	c.726C > A	
		p.(Phe242Leu)	

The mutation in *KCNH1* (c.1042G > A) has a damaging effect according to all different effect predictors tested. *STK36* has a missense mutation (c.2131 T > C), which also has damaging effects according to half of the effect predictors tested. *ZNF517* has a missense mutation (c.726C > A) predicted as disease causing by one of the effect predictors.

The *KCNH1* mutation results in a substitution of Glycine by Arginine. Same mutation is found in both isoforms of this protein: p.(Gly348Arg) in short isoform (NM_002238.3) and p.(Gly348Arg) in long isoform (NM_172362) in the ion transport domain. The p.(Gly348Arg) mutation maps to the connecting loop between helices S4-S5 as reported by Kortum et al., and exerts a strong impact on function [18].

## Discussion

We report on a male Lebanese patient in which a *de novo* missense heterozygous mutation c.1042G > A in the *KCNH1* gene led to TMBTS.

KCNH1 is a member of voltage-gated potassium channel proteins. It is recognized as an important regulator of cell proliferation in bone-marrow derived mesenchymal stem cells, and is involved in fundamental cellular and developmental processes [15, 16].

Mutations in *KCNH1* have been recently associated with TMBTS [6]. Moreover, *de novo* gain-of-function mutations in *KCNH1* have also been reported in individuals with ZLS [7].

Generally, TMBTS and ZLS can be distinguished by their characteristic phenotypic features, which include absence or dyplasia of all nails and hypertrichosis in ZLS vs hypoplasia or aplasia of only the great toe and thumb’s nails in TMBTS (Table 2). With this in mind, we considered that our patient had TMBTS. These syndromes are currently considered to be two separate entities, but their common characteristics suggest that these two syndromes may be different presentations of the same disorder. In fact, many common characteristics of patients with TMBTS and ZLS have been noted, such as, seizures, hypertrichosis, hypotonia, aplasia of nails, etc., which sometimes occur in some but not all patients (Table 2). It is noteworthy to mention that many clinical databases do not even mention TMBTS as a differential diagnosis for ZLS because of the absence of hypertrichosis, even though not all reported patients with ZLS present this characteristic.

**Table 2:** Review of all cases with the Temple-Baraitser Syndrome and a comparison to the Zimmermann-Laband syndrome characteristics

	Temple Baraitser Syndrome (TMBTS)									Zimmermann-Laband syndrome (ZLS)						
	Present Patient	Temple and Baraitser (1991) [1]	Gabbett et al. (2008) [2] or Simons et al. (2014) Patient A	Jacquinet et al. (2010) [3] Patient 1 or Simons et al. (2014) Patient D	Jacquinet et al. (2010) [3] Patient 2 or Simons et al. (2014) Patient E	Yesil et al. (2013) or Simons et al. (2014) Patient C	Shen (2015) [5] Patient 1 or Simons et al. (2014) Patient F	Shen (2015) [5] Patient 2 or Simons et al. (2014) Patient B	Total of affected patients with TMBTS with *KCNH1* mutations	Castori et al.(2013)	Zimmermann-Laband syndrome Kortüm et al. (2015) [18] *KCNH1* mutations	Zimmermann-Laband syndrome Kortüm et al. (2015) [18] *ATP6V1B2* mutations	Bramswig et al. (2015) [19] Individual 1	Bramswig et al. (2015) [19] Individual 2	Bramswig et al. (2015) [19] Individual 3	Bramswig et al. (2015) [19] Individual 4
Complicated Prgenancy	+	-	+	-	-	-	-	+		ND	ND	ND	ND	ND	ND	ND
Milestone																
Birth weight	2,700 g (60^th^ percentile)	3,370 g^a^	3,980 g (90^th^ centile)	3,590 g (50^th^ centile)	2,980 g (40^th^ centile)	3,600 g (50^th^ percentile)	7 pounds 7 ounces	3,544 g (50^th^ centile)					2,710 g	2,850 g	3,354 g	NA
Height at birth	48 cm (75^th^ percentile)		ND	ND	45 cm (10^th^ centile)	52 cm (50-75 percentile)							45 cm	50 cm	52 cm	NA
Head circumference at birth	33 cm (60^th^ percentile)	35.5 cm^a^	ND	34 cm (30^th^ centile)	33 cm (40^th^ centile)								34 cm	35 cm	NA	NA
Clinical findings																
Age (years)	0^9/12^	3^5/12^	4^4/12^	6^10/12^	1^1/12^	3^7/12^	0^9/12^	5^6/12^					14^1/12^	4^4/12^	3^9/12^	13
Consanguinity	-	-	-	-	-	-	-	-					-	-	-	-
Limbs																
Absence/hypoplasia of thumb nail	+	+	+	+	+	+	+	+ and of all fingers	8/8	52/52 Hypoplasia/aplasia of nails/phalanges	5/6 Hypoplasia/aplasia of nails	2/2 Hypoplasia/aplasia of nails	-	-	-	-
Absence/hypoplasia of hallux nail	+	+	+	+	+	+	+	+	8/8		5/6	2/2	+	+	+	+
Broad thumbs terminally	+	-	+	+	+	+	-	+	6/8	ND	0/4	ND	-	-	+	-
Thumbs; long/proximaly set	+	ND	+	+	+	+	+	+	7/7	ND	3/4	ND	+	-	-	+
Adductus deformity of distal thumb	+	ND	+	+	+	+	+	+	7/7	N D	ND	ND	ND	ND	ND	ND
Pseudoepiphysis of the thumb	-	-	+	+	+	ND	ND	ND	3/5	ND	ND	ND	ND	ND	ND	ND
Pseudoepiphysis of the great toe	-	ND	-	+	-	ND	ND	Absence of the secondary ossification center and longer great toes		ND	ND	ND	ND	ND	ND	ND
Pseudoepiphysis of the distal thumb phalanges	-	+	+	+	+	+	no but malpatterned	no but malpatterned	5/8	ND	ND	ND	ND	ND	ND	ND
Hypoplasia of distal phalanges (II-V)	-	+	+	-	+	+	+	+	6/8	52/52 Hypoplasia/aplasia of nails/phalanges	4/5 Hypoplasia/aplasia of terminal phalanges; 1 NA	2/2 Hypoplasia/aplasia of terminal phalanges	ND	ND	ND	ND
Delay in epiphyseal maturation	+	ND	ND	ND	ND	ND	ND	ND		ND	ND	ND	ND	ND	ND	ND
Neurologic																
Intellectual disability	+	+	+	+	N/A	+	+	+	7/7	21/52	6/6	2/2	+	+	+	+
Poor visual contact	+	+	ND	+	+	+	ND	ND	5/5	ND	ND	ND	ND	+	+	+
Autistic behavior	-	+	+	ND	ND	+	ND	ND	3/4	ND	ND	ND	ND	+	+	ND
Seizures	-	ND	+	+	+	+	One seizure	+	6/7	7/52	6/6	0/2 (patients ages: 22 and 5 years)	+	-	+	+
Hypotonia/motor retardation	+	+	+	+	+	+	+	+	8/8	6/52	6/6	2/2	+	+	+	+
Occipitofrontal circumference (centile)	10^th^	10^th^	25-50^th^	25-50^th^	25^th^	ND	ND	ND		ND	ND	ND	ND	ND	ND	ND
Hearing loss	-	ND	ND	ND	ND	ND	ND	ND		2/52	1/4 2 NA	1/2	ND	ND	ND	ND
Abnormal MRI findings	-	Widespread cerebral atrophy	-	-	-	Mild frontotemporal atrophy	-	-		ND	2/4 2 NA	1/1 1 NA	-	Hypoplastic corpus callosum, cystic lesion pineal gland	-	Cystic lesion pineal gland
Dysmorphic features																
Thoracic abnormalities	+	ND	ND	ND	-	ND	ND	ND		1 has Pectus carinatum and thoracic kyphosis. Others ND	1 has pectus carinatum	ND	ND	ND	ND	ND
Spine abnormalities	-	ND	ND	ND	ND	ND	ND	ND		8/52	5/6 Scoliosis	1 Scoliosis	ND	ND	ND	ND
Coarse face	+	ND	ND	ND	ND	ND	ND	ND		at least 1. Others ND	6/6	2/2	ND	ND	+	ND
Myopathic appearance	+	ND	+	+	+	+	+	+	7/7	ND	4/5	ND	+	+	+	+
Low anterior hairline	-	+	ND	ND	ND	ND	+	High anterior hairline		ND	1/6	ND	ND	ND	ND	ND
Coarse thick hair	-	ND	+	Hypertrichosis	-	+	ND	ND		Facial hypertrichosis in 8/52, body hypertrichosis in 19/52	Hypertrichosis 3/6	Marked hypertrichosis 2/2	+	+	+	+
Flat forehead	Bulging	+	+	+	+	+	ND	ND	5/6	ND	ND	ND	Prominent	ND	Broad and prominent	ND
Mild hypertelorism	+	+	-	+	+	+	+	+	7/8	6/52	4/5	ND	+	+	+	+
Epicanthal folds	+	-	+	-	+	+	-	-	4/8	ND	1/6	ND	-	+	+	-
Broad depressed nasal bridge	+	+	+	+	+	+	+	+	8/8	ND	3/4 depressed 5/5 broad	ND	+	+	+	Only broad
Short columella	+	-	+	+	+	+	-	+	6/8	ND	4/4	ND	+	+	+	-
Long philtrum	+	+	+	+	+	+	+	+	8/8	ND	2/6 1 short philtrum	1/2	ND	ND	ND	ND
Thick/full vermillion border of upper lip	-	ND	+	+	+	Upper and lower lip	Upper and lower lips	Tented vermilion of upper lip and everted thick vermilion of the lower lip	5/7	27 thick lips/macrostomia	5/6	ND	+	+	+	+
Broad mouth with downturned corners	+	ND	+	+	+	+	+	+	7/7	ND	4/4	ND	+	+	+	+
Gingival enlargement	+	-	-	-	-	-	-	-	1/8	52/52	5/6	2/2	+	+	+	+
Narrow and high palate	+	ND	+	ND	ND	ND	+	ND		11/52	ND	ND	ND	ND	ND	ND
Inverted nipples	Widely spaced	ND	ND	ND	ND	Widely spaced	ND	ND		ND	ND	ND	ND	ND	ND	ND
Systemic manifestations																
Gastrointestinal symptoms	Constipation	Early feeding difficulties with recurrent vomiting	-	Severe gastroesophageal reflux in the neonatal period	ND	Constipation	-	Constipation		ND	3/6 have gastroesophageal reflux and/or constipation	ND	Constipation	Slight feeding problem	Constipation	Severe feeding problem
Small genitalia/endocrine anomalies	-	ND	ND	ND	ND	+	ND	ND		3/52 abnormal genitalia	ND	1 has macroorchidism	ND	ND	ND	ND
Cardiovascular system anomalies	-	ND	-	ND	ND	Atrial septal defect and mild pulmonary stenosis	ND	ND		6/52	ND	ND	-	ND	-	Open ductus bodalli

*Abbreviations:* +, present; −, absent; *NA* not analyzed, *ND* not documented, *N/A* not applicable, *MRI* magnetic resonance imaging

^a^ no standard deviation noted

Interestingly, the same mutation (c.1042G > A) identified in our patient has never been reported with TMBTS, but was previously detected in patients with ZLS (patient 7 in Abo-Dalo et al. or subject 3 in Kortüm et al.) [17, 18]. This substitution leads to a gain of function effect and mutants carrying this mutation exhibit an accelerated channel activation and a slower deactivation [18]. Along with the previously identified p.(Ile494Val) misense variant in *KCNH1*, which was shared among individuals with TMBTS and ZLS, the genetic defect identified in our patient, i.e., p.(Gly348Arg) was found in patients bearing different phenotypes and thus supposedly different syndromes. This provides stronger evidence that both syndromes clearly overlap and could be a phenotypic continuum. In fact, the mutation c.1042G > A was found in a patient with ZLS who does not present with hypertrichosis, similar to the patient reported herein. However, the patient had in addition, aplasia of all nails of hands and feet, thoracic scoliosis, and infrequent seizures, which were not present in our patient who had a delay in epiphyseal maturation, (Table 3) a feature never reported before in both entities, and gingival enlargement. The latter is a characteristic not reported previously in TMBTS affected individuals, however it is a frequent feature in patients with mutations of *KCNH1* (Bramswig et al.). Genetic modifiers, possibly involving the Na^+^ and Ca^2+^ channels, might block the KCNH1 channels and result in the gingival enlargement as it is observed in individuals treated with Na^+^ blocker phenytoin or Ca^2+^ channel blocker nifedipine [18].

**Table 3:** Clinical comparison between the patient here described with TMBTS and the patient described by Kortüm et al. (subject 3)

	Patients having the p.(Gly348Arg) mutation	
	Present patient	Subject 3 in Kortüm et al. (2015)
Gender	M	F
Complicated Pregnancy	+	ND
Milestone		
Birth weight	2.700 g (60^th^ percentile)	3,290 g (39 weeks) (54^th^ percentile)
Height at birth	48 cm (75^th^ percentile)	55 cm (99^th^ percentile)
Head circumference at birth	33 cm (60^th^ percentile)	ND
Clinical findings		
Age (years)	0^9/12^	19
Consanguinity	-	ND
Limbs		
Absence of nails	Nails of thumb and hallux	Nails of hands and feet
Broad, long thumbs terminally	+	ND
Adductus deformity of distal thumb	+	ND
Hypoplasia of terminal phalanges of hands and feet	Nearly absent	+
Delay in epiphyseal maturation	+	ND
Neurologic		
Intellectual disability	+	Severe
Poor visual contact	+	ND
Seizures	-	Started in adolescence
Hypotonia/motor retardation	+	+
Hearing loss	-	-
Abnormal MRI findings	-	NA
Dysmorphic features		
Thoracic abnormalities	+	Thoracic scoliosis
Coarse face	-	+
Myopathic appearance	+	ND
Hypertrichosis	-	-
Coarse thick hair	-	-
Flat forehead	Bulging	ND
Mild hypertelorism	+	ND
Epicanthal folds	+	ND
Broad depressed nasal bridge	+	ND
Short columella	+	ND
Long philtrum	+	ND
Thick vermillion border of upper lip	-	ND
Broad mouth with downturned corners	+	ND
Gingival enlargement	+	Noticed in childhood prior anticonvulsant treatment
Central incisors	+	+
Narrow and high palate	+	ND
Inverted nipples	Widely spaced	ND
Systemic manifestations		
Gastrointestinal symptoms	Constipation	ND
Small genitalia/endocrine anomalies	-	Solitary renal cyst
Cardiovascular system anomalies	-	ND

*Abbreviations:* +, present; −, absent; *ND* not documented

On the other hand, the patient reported by Kortum et al., developed seizures in adolescence, therefore one could speculate a late occurence of epilepsy in the patient described here with the same mutation. Yet, Bramswig et al. described 3 individuals presenting with an identical *KCNH1* variant but with different clinical features with regard to epilepsy [19]. Consequently, the presence of a pathogenic *KCNH1* variant alone could not allow for a prediction of occurence of epileptic seizure.

Other genetic modifiers could be responsible for the observed differences in clinical phenotype. We looked deeper at the results of the WGS and noticed mutations in two other genes *STK36* and *ZNF517*, which were classified in some databases as possibly damaging. However, their significance remains to be elucidated. Recently, *de novo* mutations in *STK36* have been identified in patients with epileptic encephalopathies [20]. Although our patient who has missense mutation in *STK36* does not present with epilepsy at present, he might develop it in adolescence as in patient 3 in Kortum et al. Thus, concordant to previous reports, our data supports the evidence that the mutated *KCNH1* is a major cause of TMBTS and ZLS, while other genes can act as disease modifying roles. Understanding the molecular mechanisms by which these genes exert disease modifying roles might help in the better understanding of the pathogenesis of these syndromes.

Finally, both ZLS and TMBTS patients with *KCNH1* mutations show similar phenotypes. Nevertheless, two other ZLS patients were also described with mutations in the *ATP6V1B2* gene that encodes a component of the vacuolar ATPase (V-ATPase). These mutations present a more pronounced phenotype characterized mostly by hypertrichosis and a coarser facial phenotype (Table 2). But due to the limited number of individuals described, a conclusion about whether probands with mutations involving *ATP6V1B2* lead to a more severe syndrome might not be accurate. On the other hand, Kortüm et al. screened a cohort of 24 ZLS patients, of which only 8 had mutations in *KCNH1* and *ATP6V1B2* suggesting further the genetic heterogeneity in the ZLS disorder [18].

## Conclusions

In summary, this study shows that the same *KCNH1* mutation can lead to both ZLS and TMBTS. The phenotypic variability could be the result of a modifier gene or genes, and identification of such genes would be of great importance. A careful analysis of genetic polymorphisms in various loci should be taken into consideration for clinical diagnosis. Further investigations are needed to confirm if *ATP6V1B2* mutations lead to a more severe phenotype.

## Abbreviations

ID, intellectual disability; TMBTS, Temple-Baraitser syndrome; WGS, whole genome sequencing; ZLS, Zimmermann-Laband syndrome.

## References

[1] Temple IK, Baraitser M (1991). Severe mental retardation and absent nails of hallux and pollex. Am J Med Genet.

[2] Gabbett MT, Clark RC, McGaughran JM (2008). A second case of severe mental retardation and absent nails of hallux and pollex (Temple-Baraitser syndrome). Am J Med Genet A.

[3] Jacquinet A, Gérard M, Gabbett MT, Rausin L, Misson J-P, Menten B, et al. Temple-Baraitser syndrome: a rare and possibly unrecognized condition. Am J Med Genet A. 2010;152A:2322–6. 10.1002/ajmg.a.33574 20683999

[4] Yesil G, Guler S, Yuksel A, Alanay Y (2014). Report of a patient with Temple-Baraitser syndrome. Am J Med Genet A.

[5] Shen JJ (2015). Two cases of Temple-Baraitser syndrome: natural history and further delineation of the clinical and radiologic phenotypes. Clin Dysmorphol.

[6] Simons C, Rash LD, Crawford J, Ma L, Cristofori-Armstrong B, Miller D, et al. Mutations in the voltage-gated potassium channel gene KCNH1 cause Temple-Baraitser syndrome and epilepsy. Nat Genet. 2015;47:73–7. 10.1038/ng.3153 25420144

[7] Castori M, Valiante M, Pascolini G, Leuzzi V, Pizzuti A, Grammatico P (2013). Clinical and genetic study of two patients with Zimmermann-Laband syndrome and literature review. Eur J Med Genet.

[8] Miller SA, Dykes DD, Polesky HF (1988). A simple salting out procedure for extracting DNA from human nucleated cells. Nucleic Acids Res.

[9] Li H, Durbin R (2009). Fast and accurate short read alignment with Burrows-Wheeler Transform. Bioinformatics.

[10] DePristo M, Banks E, Poplin R, Garimella K, Maguire J, Hartl C, et al. A framework for variation discovery and genotyping using next-generation DNA sequencing data. Nat Genet. 2011;43:491–8. 10.1038/ng.806 PMC3083463 21478889

[11] Adzhubei IA, Schmidt S, Peshkin L, Ramensky VE, Gerasimova A, Bork P, et al. A method and server for predicting damaging missense mutations. Nat Methods. 2010;7:248–9. 10.1038/nmeth0410-248 PMC2855889 20354512

[12] Kumar P, Henikoff S, Ng PC (2009). Predicting the effects of coding non-synonymous variants on protein function using the SIFT algorithm. Nat Protoc.

[13] Schwarz JM, Rodelsperger C, Schuelke M, Seelow D (2010). Mutation Taster evaluates disease-causing potential of sequence alterations. Nat Methods.

[14] Shihab HA, Gough J, Cooper DN, Day IN, Gaunt TR (2013). Predicting the functional consequences of cancer-associated amino acid substitutions. Bioinformatics.

[15] Ouadid-Ahidouch H, Le Bourhis X, Roudbaraki M, Toillon RA, Delcourt P, Prevarskaya N (2001). Changes in the K+ current-density of MCF-7 cells during progression through the cell cycle: possible involvement of a h-ether.a-gogo K+ channel. Receptors Channels.

[16] Hemmerlein B, Weseloh RM, Mello de Queiroz F, Knötgen H, Sánchez A, Rubio ME, et al. Overexpression of Eag1 potassium channels in clinical tumours. Mol Cancer. 2006;5:41. 10.1186/1476-4598-5-41 PMC1621079 17022810

[17] Abo-Dalo B, Roes M, Canún S, Delatycki M, Gillessen-Kaesbach G, Hrytsiuk I, et al. No mutation in genes of the WNT signaling pathway in patients with Zimmermann-Laband syndrome. Clin Dysmorphol. 2008;17:181–5. 10.1097/MCD.0b013e3282f2514c 18541964

[18] Kortüm F, Caputo V, Bauer CK, Stella L, Ciolfi A, Alawi M, et al. Mutations in KCNH1 and ATP6V1B2 cause Zimmermann-Laband syndrome. Nat Genet. 2015;47:661–7. 10.1038/ng.3282 25915598

[19] Bramswig NC, Ockeloen CW, Czeschik JC, van Essen AJ, Pfundt R, Smeitink J, et al. ‘Splitting versus lumping’: Temple-Baraitser and Zimmermann-Laband Syndromes. Hum Genet. 2015;134:1089-97. 10.1007/s00439-015-1590-1 26264464

[20] Allen AS, Berkovic SF, Cossette P, Epi4K Consortium; Epilepsy Phenome/Genome Project (2013). De novo mutations in epileptic encephalopathies. Nature.

